# Reply to Najjar, R.S. Myths of the Low-Carbohydrate Diet “Myths”. Comment on “Teicholz et al. Myths and Facts Regarding Low-Carbohydrate Diets. *Nutrients* 2025, *17*, 1047”

**DOI:** 10.3390/nu18213597

**Published:** 2026-10-30

**Authors:** Nina Teicholz, Steven M. Croft, Ignacio Cuaranta, Mark Cucuzzella, Mariela Glandt, Dina H. Griauzde, Karen Jerome-Zapadka, Tro Kalayjian, Kendrick Murphy, Mark Nelson, Catherine Shanahan, Jodi L. Nishida, Robert C. Oh, Naomi Parrella, Erin M. Saner, Shebani Sethi, Jeff S. Volek, Micalla Williden, Susan Wolver

**Affiliations:** 1 Independent Researcher, New York, NY 10024, USA; 2 Independent Researcher, Houston, TX 77074, USA; 3 Independent Researcher, Rosario 2000, Santa Fe, Argentina; 4 Department of Family Medicine, West Virginia University School of Medicine, Shepherdstown, WV 26506, USA; 5 OwnaHealth, New York, NY 10006, USA; 6 Department of Internal Medicine, University of Michigan School of Medicine, Ann Arbor, MI 48105, USA; 7 Trajectory Health Partners, Beaver Falls, PA 15010, USA; 8 Toward Health, Blauvelt, NY 10913, USA; 9 Western North Carolina VA Health Care System, Asheville, NC 28805, USA; 10 Independent Researcher, Chicago, IL 60174, USA; 11 Independent Researcher, Orlando, FL 32803, USA; 12 The Keto Prescription Clinic, Honolulu, HI 96816, USA; 13 Department of Medicine, Stanford University School of Medicine, Palo Alto, CA 94305, USA; 14 Department of Family and Preventive Medicine, Rush Medical College, Chicago, IL 60612, USA; 15 Department of Family & Community Medicine, Wake Forest University School of Medicine, Winston-Salem, NC 27104, USA; 16 Department of Psychiatry and Behavioral Sciences, Stanford University School of Medicine, Stanford, CA 94305, USA; 17 Department of Human Sciences, The Ohio State University, Columbus, OH 43210, USA; 18 Independent Researcher, Auckland 0618, New Zealand; 19 Department of Internal Medicine, Virginia Commonwealth University School of Medicine, Richmond, VA 23298, USA

We note that the names of the commenters were not disclosed to us when we prepared this response.

The commenters [1] assert that our view on saturated fats contradicts the “established scientific consensus”, citing a review by the American Heart Association (AHA). A broad official consensus on saturated fats has indeed existed since the early 1960s, as reflected in dietary guidelines globally. However, the last 15 years have seen a quiet renaissance of scientific thinking on these fats. A paper in *PNAS Nexus* reported in 2022 that 20 review articles “by independent teams of scientists from around the world [that] have now challenged the scientific basis” for the advice to “support limits on saturated fats as a measure of protection against heart disease” [2]. At least two additional reviews have since concluded the same [3,4]. Thus, no scientific consensus on this topic can currently be claimed. 

The widespread finding from these reviews is that in clinical trials whereLDL cholesterol is lowered by replacing saturated fats with seed oils, there is no effect on cardiovascular or total mortality. Many mechanisms have been proposed to explain the lack of association between LDL-lowering and mortality observed in dietary trials. One is that saturated fats reliably raise HDL, the “good” cholesterol, which may counteract their effects on LDL.

Most official statements on saturated fats have not considered this new ‘renaissance’ in thinking, and some have omitted the important mortality data from RCTs. For instance, the AHA review did not evaluate the clinical trial data on “hard” outcomes, such as heart attacks and mortality, which are a more reliable form of evidence than intermediary markers such as LDL cholesterol. In addition, the AHA has a significant conflict of interest, as it authored the original policy advising the public to reduce saturated fat to prevent heart disease, in 1961 [5]. The commenters’ appeal to authority is a logical fallacy because science is meant to be based on empiricism, not expert hierarchy. 

On the topic of refined versus unrefined carbohydrates, we acknowledge the evidence that refined carbohydrates such as flours and sugars have both a higher glycemic index and a higher glycemic load (gram for gram), and thus have different cardiometabolic impacts than do complex carbohydrates. However, our paper states at the outset that we intended to focus on results from controlled clinical trials, because these are the highest-quality form of evidence. As cited extensively throughout our paper, these data demonstrate that reductions in *total* carbohydrates, irrespective of the degree of refinement, have demonstrated the greatest benefit for chronic disease reversal. We look forward to more rigorous research on the question of how carbohydrate refinement affects metabolic disease to further elucidate this issue with confidence. 

The commenters state that it is “unequivocal” that certain carbohydrate-rich foods reduce CVD risk and that “replacing calories from saturated fats with whole grains reduces the risk of CVD”, yet both these claims rely exclusively on observational studies, a low-quality type of evidence that cannot reliably establish cause-and-effect relationships [5]. Further, they are contradicted by the largest-ever observational study, Prospective Urban Rural Epidemiology (PURE), which found that higher saturated fat consumption is associated with significantly *lower* mortality [6].

The claim that a reduction in blood glucose does not indicate type 2 diabetes reversal is contradicted by the most prominently published definition of “reversal” itself, from a 2022 Consensus Report cited in our paper, stating that reversal is “the process of returning to glucose levels below those diagnostic of diabetes” without glucose-lowering drugs, as measured by HbA1c, average blood sugar. Another Consensus Report, by the American Diabetes Association in 2019, confirms that a reduction in carbohydrate, not dietary fat, “has demonstrated the most evidence for improving glycemia [blood sugar control]” [7]. If lipotoxicity were indeed the cause of insulin resistance, one would expect to see the reversal of type 2 diabetes on a low-fat diet. However, to substantiate this point, the commenters cite a single 16-day trial on 20 men, from 1979, which resulted in only “slightly” lowered plasma glucose values among the men on a low-fat, high-carbohydrate diet (HbA1c is not reported). The commenters omit the far more significant National Institutes of Health-funded clinical trials, testing more than 50,000 people for 1–7 years, which did not find that a carbohydrate-rich diet could reverse or prevent type 2 diabetes [8,9,10]. The only nutritional approaches that have demonstrated type 2 diabetes reversal are a very-low-calorie, formula-based diet and one low in carbohydrates [11]. 

We find it lamentable that “experts” continue to be so resistant to the science on low-carbohydrate diets and to denigrate a nutritional approach that has proven to be life-saving for so many. 

## Data Availability

No new data were created or analyzed in this study. Data sharing is not applicable to this article.
